# Sporadic Medullary Thyroid Carcinoma: Towards a Precision Medicine

**DOI:** 10.3389/fendo.2022.864253

**Published:** 2022-03-29

**Authors:** Antonio Matrone, Carla Gambale, Alessandro Prete, Rossella Elisei

**Affiliations:** Department of Clinical and Experimental Medicine, Endocrine Unit, University Hospital of Pisa, Pisa, Italy

**Keywords:** medullary thyroid cancer, calcitonin, tyrosine kinase inhibitors, RET gene, highly selective RET inhibitors, immunotherapy

## Abstract

Medullary thyroid carcinoma (MTC) is a neuroendocrine malignant tumor originating from parafollicular C-cells producing calcitonin. Most of cases (75%) are sporadic while the remaining (25%) are hereditary. In these latter cases medullary thyroid carcinoma can be associated (multiple endocrine neoplasia type IIA and IIB) or not (familial medullary thyroid carcinoma), with other endocrine diseases such as pheochromocytoma and/or hyperparathyroidism. RET gene point mutation is the main molecular alteration involved in MTC tumorigenesis, both in sporadic and in hereditary cases. Total thyroidectomy with prophylactic/therapeutic central compartment lymph nodes dissection is the initial treatment of choice. Further treatments are needed according to tumor burden and rate of progression. Surgical treatments and local therapies are advocated in the case of single or few local or distant metastasis and slow rate of progression. Conversely, systemic treatments should be initiated in cases with large metastatic and rapidly progressive disease. In this review, we discuss the details of systemic treatments in advanced and metastatic sporadic MTC, focusing on multikinase inhibitors, both those already used in clinical practice and under investigation, and on emerging treatments such as highly selective RET inhibitors and radionuclide therapy.

## Introduction

Medullary thyroid carcinoma (MTC) is a neuroendocrine malignant tumor originating from parafollicular C-cells producing calcitonin (CTN) a highly sensitive biomarker used for the diagnosis and the follow-up of MTC (1). Moreover, parafollicular C-cells can produce several other peptides (2) among which carcinoembryonic antigen (CEA) can be also used as biomarker of tumor burden. MTC shows the same distribution in male and females and the median age at diagnosis is usually in the fourth and fifth decades of life (3–5). The prevalence of MTC is highly variable according to the different studies considered. Nevertheless, it accounts for 0.4-1.4% of all thyroid nodules, about 2% of all thyroid cancers and about 0.14% of all thyroids of subjects submitted to autopsy (6–8). Thus, National Health Institute (NIH) included MTC in the list of rare disease.

Most MTC are sporadic (about 75%) but the remaining (25%) are hereditary. In the latter, due to inherited (autosomal dominant) REarranged during Transfection (*RET*) protooncogene alteration (9–12), MTC can be associated (multiple endocrine neoplasia type IIA and IIB – MEN IIA and MEN IIB) or not (familial medullary thyroid carcinoma – FMTC), with other endocrine neoplasia such as pheochromocytoma (PHEO) and/or hyperparathyroidism due to parathyroid hyperplasia or multiple adenomatosis (PTHAd) (13). MTC in children is extremely rare and it can be found almost exclusively in inherited cases of MEN II (14–17).

MTC can potentially spread both by lymphatic and hematic vessels, for this reason its clinical behavior is worse than differentiated thyroid carcinoma (DTC) but without reaching the aggressiveness of the anaplastic ones (ATC) (18, 19). The 10-year disease specific mortality varies from 13.5 to 38% (20, 21) and the 10-year survival could decrease up to 50% (22–27).

Several factors were related to the disease specific mortality, such as advanced stages and age at the diagnosis (21, 28). An early diagnosis associated to intrathyroidal tumors have the better survival rate, up to 90% at 35 years (27, 29). Since the aim of this review is to show the therapeutic possibilities of sporadic advanced cases of MTC, we did not discuss familial cases.

## Clinical Presentation

The most common clinical presentation of a sporadic MTC is a thyroid nodule, single or in a multinodular goiter. However, the diagnostic workup for the diagnosis of MTC is still controversial. Several debates are still ongoing about the routine use of serum CTN in the evaluation of thyroid nodules (1), and in the diagnosis and treatment of low stage MTC (30).

Indeed, if thyroid function assessed by the measurement of free triiodothyronine (fT3), free thyroxine (fT4) and thyroid stimulating hormone (TSH) is usually in the normal range, the presence of elevated values of serum CTN, and CEA particularly in advanced cases, sometimes represents the first suspicion of the MTC, thus requiring further diagnostic procedures (31). Moreover, CTN and CEA are the biochemical markers of MTC patients, both in the diagnosis and after surgery, even during local or systemic treatments.

Differently from DTC, the sensitivity of neck ultrasonography in detecting MTC is quite low, also when the 5 main ultrasonographic risk stratification systems were applied (32). Moreover, fine needle aspiration cytology (FNAC) showed low sensitivity in identifying MTC (33–35).

Lymph nodes metastases are often detected at the time of diagnosis and distant metastases are already present in about 10-20% of MTC patients, according to the different series (36, 37). Symptoms such as diarrhea and/or flushing syndrome could occur in advanced metastatic cases, associated with high levels of serum CTN (29, 38, 39).

## Initial Treatment

Clinical presentation of MTC plays a key role in the decision making of the initial treatment to be performed. In the absence of latero-cervical lymph nodes metastases, total thyroidectomy with prophylactic/therapeutic central compartment lymph nodes dissection is the standard of care. When latero-cervical lymph nodes metastases are detected before or during surgery, a localized compartment lymph node dissection is suggested (1). Primary tumor and lymph nodes metastases should be removed also if distant metastases are already present (1). In cases of locally invasive tumors compromising vital structures in the neck where surgery is not feasible, additional treatments should be performed (40, 41).

## Other Treatments

**Figure 1:**
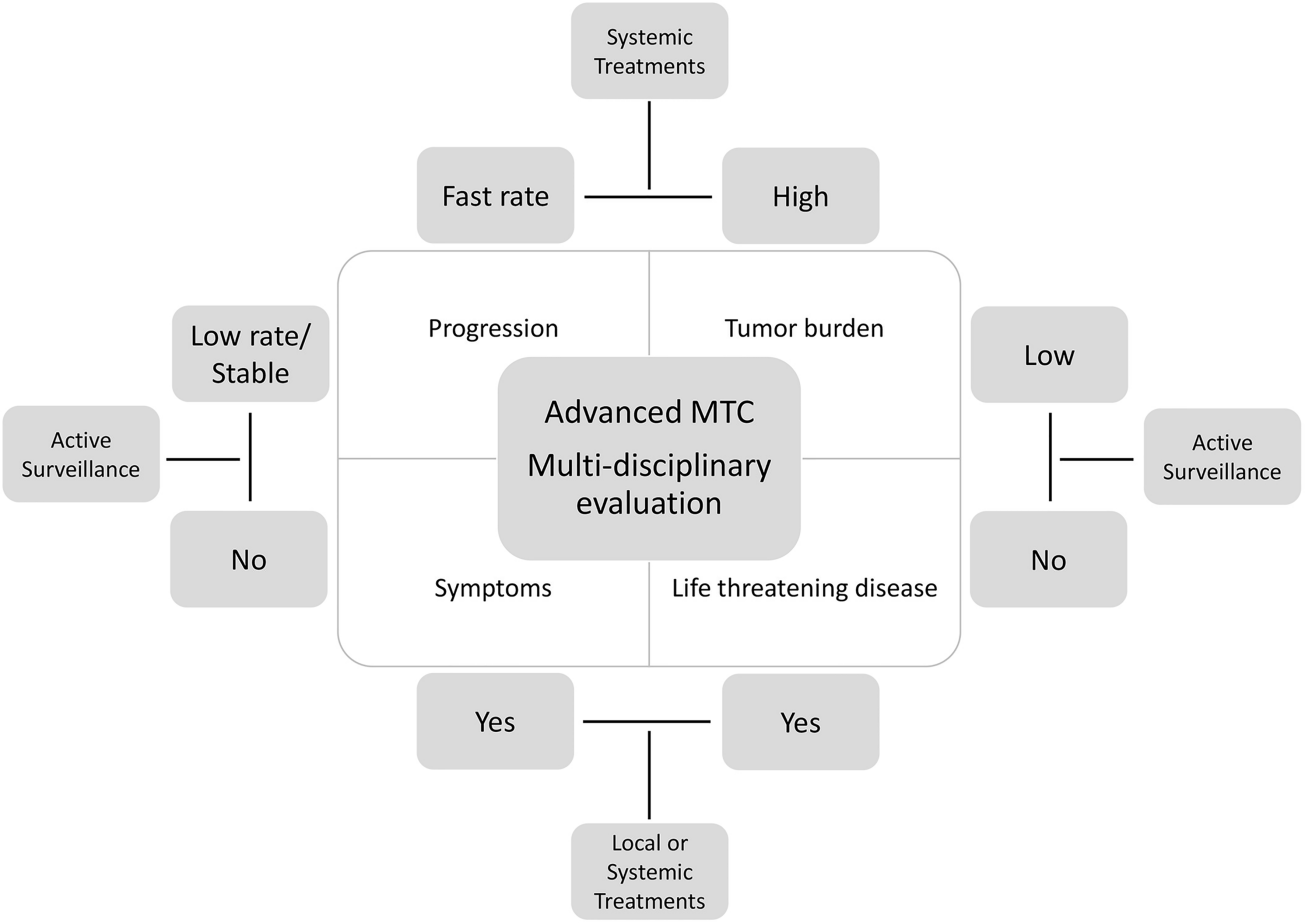
Algorithm for the management of advanced MTC: not all cases require a systemic therapy. Active surveillance or local treatments can be appropriate in some cases according to the rate of growth, tumor burden, symptoms, and life-threatening disease.

Tumor burden and progression rate are determinant in the decision making to start a systemic treatment (**Figure 1**). Progression rate should be evaluated according to the Response Evaluation Criteria in Solid Tumors (RECIST 1.1) (42).

RECIST aimed to be objective and reproducible criteria and takes into account all measurable lesions up to a maximum of five lesions per organ and ten lesions in total. Target lesions should be selected based on the size (those with longest diameter) and if can be accurate measured over time. Progressive disease (PD) is defined by at least a 20% increase in the sum of the longest diameter of target lesions, or the appearance of one or more new lesions. All other lesions identified by the investigator, and not classified as target, should be defined as non-target and should be recorded and monitored during the follow-up.

When a progressive disease is documented accordingly, therapeutic interventions should be planned.

A small tumor burden and/or a slow progression rate, in the absence of symptoms or high risk of local complications are indications to active surveillance. However, in case of localized progression in a single lesion or in multiple lesions but in the same organ, the use of local therapies is advocated (1).

## When Starting Systemic Treatments

Several considerations should be taken in patients with advanced metastatic MTC before starting a systemic treatment (43) (**Figure 1**). Since not all patients with metastatic disease will progress over time or will die from the disease, these patients should be carefully evaluated at 6 months intervals by clinical and biochemical evaluation. The dosage of serum CTN and CEA and calculation of their doubling time (DT) should be performed at each visit. Bad prognoses were reported when DT of CTN and/or CEA were less than 0.5-1 year (44). Moreover, serum carbohydrate antigen 19.9 (Ca19.9) particularly in advanced MTC, represents a poor prognostic factor for survival (45).

In these cases, imaging evaluation should be intensified. Total body computed tomography (CT) scan with i.v. contrast medium is the best currently available and reproducible method to evaluate the site of metastatic spread and the progression of disease. Neck US is highly sensitive for detecting lymph node metastases, magnetic resonance imaging (MRI) is useful in defining liver metastases, while bone scintigraphy is used bone metastases, particularly if osteoblastic.

In recent years, several radiotracers have been used for Positron Emission Tomography/Computed Tomography (PET/CT) scanning in MTC patients, such as 18F-fluorodeoxyglucose (18F-FDG), 18F-dihydroxyphenylalanine (18F-DOPA) able to target the amine decarboxylation pathway and 68Ga DOTA-labeled somatostatin analogues (46–48). Although functional imaging, particularly 18F-DOPA PET/CT, was extensively used in the diagnostic work-up of advanced MTC, to date, limited impact on therapeutic management was demonstrated (49).

In case of large tumor burden with multiple distant metastatic sites and fast rate of progression (<12-14 months), a systemic treatment should be started based on RECIST criteria as well as on clinical judgment. Waiting for progression of disease based on RECIST criteria alone could delay the initiation of treatment too much (50, 51).

Moreover, in advanced cases, the patients frequently experience diarrhoea which can significantly impair their quality of life. Diarrhoea is often related to the tumor burden: the greater the tumor burden, the more frequent and intense the diarrhoea. In these symptomatic cases, since the diarrhoea is linked to the tumor burden, starting a systemic treatment could be a reasonable option.

## Inside the Clinical Trials

The old paradigm of using cytotoxic chemotherapy for MTC is largely outdated due to the low efficacy of these therapies in treating advanced metastatic MTC (52). For this reason, during the years, several clinical trials with multikinase inhibitors (MKI) and, more recently, with highly selective RET inhibitors were designed. Both these types of drugs are considered cytostatic and not cytotoxic. Cytotoxic drugs usually kill the cells and determines tumor shrinkage, whereas cytostatic drugs inhibit tumor growth without having a direct cytotoxic effect. However, also if the killing effect on the tumor cells is not direct, kinase inhibitors can be considered directly cytostatic and indirectly cytotoxic. Angiogenesis is the growth of new blood vessels necessary to provide oxygen, nutrients, hormones, growth factors, proteolytic enzymes and allow the metastasization of tumor cells to distant sites (53). This process is initiated and carried on by several pro-angiogenic factors of which vascular endothelial growth factor (VEGF) and its receptor (VEGF-R) are predominant (54). MTC is a highly vascularized tumor and overexpression of VEGF and VEGFR were demonstrated in MTC samples (55). Interestingly, angiogenesis was more intense in *RET*-mutant MTC (56). This is the first rationale for using MKI, also called anti-angiogenic drugs, in the treatment of advanced MTC. Conversely, the higher potency of inhibition of RET, in *RET*-mutant MTC is the key point of the efficacy of the highly selective RET inhibitors.

Since the first 2 phase III clinical trials (57, 58) testing efficacy of MKI vandetanib and cabozantinib are designed for cytostatic and not cytotoxic drugs, the primary endpoint of these studies was usually represented by the evaluation of the progression-free survival (PFS – defined as the time from first time of taking the drug to disease progression or death, whichever occurred first). Conversely the evaluation of the overall survival (OS) needs of more follow-up time and for this reason is usually a secondary end point. Several other parameters were considered to evaluate the efficacy of the treatment, particularly in the recent phase I/II study involving patients treated with selpercatinib and pralsetinib (59, 60). Objective response rate (ORR - the proportion of patients achieving a complete or partial response per RECIST 1.1) is the most widely used. Other secondary endpoints are the duration of response (DOR - the time from first tumor response until disease progression or death), clinical benefit rate (proportion of patients with a confirmed complete or partial response or stable disease of ≥16 weeks), disease control rate (proportion of patients with a complete or partial response or stable disease).

In this review we highlight the key points of the systemic treatment of advanced metastatic MTC providing an overview of the treatments developed in the last years, from MKI to the highly selective RET inhibitors, up to the radionuclide therapies.

## Multikinase Inhibitors

MKIs are drugs able to block the activity of several tyrosine kinase receptors (TKRs), involved in cell growth, differentiation, and angiogenesis. TKRs are upstream of the two main signalling pathways involved in cell proliferation: the mitogen-activated protein (MAP) kinase/extracellular signal-regulated pathway (ERK) and the phosphatidylinositol-3-kinase (PI3K)/AKT and the mammalian target of rapamycin (mTOR) pathway. Mutations or overexpression of TKRs are frequently detected in cancer cells (61, 62), leading to oncogenic transformation and tumoral progression. Therefore, the implementation of MKIs drugs in clinical practice determined important clinical implications in many human cancers such as leukaemia (63), renal carcinoma (64, 65) and hepatocellular carcinoma (66, 67). Alike, MKIs had extensive applications in thyroid oncology, both in the treatment of radioactive iodine-refractory differentiated thyroid carcinoma (68, 69) and in advanced medullary carcinoma (57, 70).

MTC cells can harbour several driver mutations, the most common involving *RET* and *RAS* genes. In about 15-20% of the MTC cases (1), mutations of the *RET* gene could be inherited. In these cases, point mutations in cysteine or tyrosine-rich domains of seven exons (8, 10, 11, 13, 14, 15 and 16) of *RET* oncogene (71, 72), are responsible for the familial forms of MEN IIA, the most frequent in codon 634 (73), MEN IIB, the most frequent in codon 918 (9) and FMTC. However, 40-70% of sporadic MTC cases (74) harboured a somatic *RET* mutation, either point mutations or indels, and more advanced is the case, more frequently *RET^M918T^* mutation is found (75).

*RAS* mutations, prevalently *H-* and *K-RAS*, are somatic point mutations found in about 20% of sporadic MTCs and are mutually exclusive with *RET* mutations (76). However, about 20% of the remaining MTC cases are still orphan of a driver mutation (76).

Over the years, several MKIs have been tested in the treatment of advanced MTC. In most of cases, the use of MKIs, such as motesanib (77), sorafenib (78) and pazopanib (79), was more effective in stabilizing the disease avoiding the progression, than in decreasing tumour burden. Also, imatinib was tested in MTC but showed no objective responses and had a considerable toxicity (80).

To date, only two MKIs have been approved by the Food and Drug Administration (FDA) and the European Medicine Agency (EMA) for the treatment of advanced MTC: vandetanib and cabozantinib.

### Vandetanib

Vandetanib (ZD6474) is a multikinase inhibitor, with high affinity for VEGFR-2 and VEGFR-3 (81), it also blocks the activity of EGFR (82) and the active conformation of both wild type and mutant *RET* (83, 84) (**Table 1**).

**Table 1:** IC_50_ of the main drugs used in the treatment of advanced MTC against the most common cell membrane receptors (81, 85–89).

	Half-life	RET^WT^	RET^V804L^	RET^V804M^	RET^M918T^	EGFR	MET	VEGFR2
Anlotinib	116 hours	/	/	/	/	>2000	>2000	0.2
Cabozantinib	55 hours	5.2	45	162	8	/	1.3	0.035
Lenvatinib	28 hours	0.19	10.6	5.4	1.4	>2000	/	2.3-4.7
Selpercatinib	32 hours	0.4	0.42	0.8	0.7	/	/	100
Pralsetinib	14.7 hours	0.4	0.3	0.4	0.4	/	/	35
Vandetanib	19 days	130	3597	726	7	0.5	/	4

IC_50_ (half maximal inhibitory concentration) indicates the concentration of drug needed to inhibit 50% of target activity and is commonly used to show the potency of a drug against a target.

Graphical legend - IC_50_ <5 nM (green), IC_50_ 5-50 nM (yellow), IC_50_ 50-200 nM (orange), IC_50_ >200 nM (red): the lower the IC_50_, the higher the potency.

RET, Rearranged During Transfection; EGFR, Epidermal Growth Factor Receptor; MET, mesenchymal-epithelial transition proto-oncogene; VEGFR2, Vascular Endothelial Growth Factor Receptor 2.

Vandetanib has been approved for the treatment of aggressive and symptomatic, unresectable, locally advanced, or metastatic MTC, after the results of the phase III ZETA trial (57) (ClinicalTrials.gov Identifier NCT00410761).

ZETA trial started in 2006, and it was a randomized, placebo-controlled, double-blind phase III trial with the aim to evaluate the efficacy and safety of vandetanib in adult patients with unresectable locally advanced or metastatic familial or sporadic MTC, compared to placebo. The primary endpoint was the PFS. The patients enrolled (n=331) were randomized in a 2:1 ratio to receive vandetanib 300 mg/daily (n = 231) or placebo (n = 100), up to disease progression. PFS was significantly longer in vandetanib compared to placebo group (30.5 vs 19.3 months; HR = 0.46; 95%CI: 0.31-0.69; *p <*0.001). Although no significant difference was observed in OS, vandetanib was associated with significant improvements in other secondary endpoints (ORR, disease control rate at 24 weeks, DOR, reductions in CTN and CEA levels). Since in the ZETA trial patients were enrolled both for advanced progressive disease or symptoms related to the disease, in a recent *post-hoc* analysis (90), the whole study population of the ZETA trial was divided into 4 groups: 1) patients who had no progression and no symptoms related to the disease, 2) patients with symptoms only, 3) patients who progressed only, 4) patients who had progressed, and experienced symptoms related to the disease. Median PFS was significantly longer in patients treated with vandetanib compared to placebo, in group 2 (22.43 vs 9.68 months; HR = 0.41; 95%CI: 0.17-; p = 0.05) and 4 (21.43 vs 8.40 months; HR = 0.43; 95%CI: 0.28-0.64; *p* < 0.0001). Conversely, in group 1 and 3, no difference was highlighted in patients treated with vandetanib and placebo. Therefore, the benefit of the treatment was particularly demonstrated in those patients who experienced progressive disease associated to symptoms.

The efficacy and safety of vandetanib was assessed also in studies outside clinical trials. A multicenter French study showed that the median PFS was 16.1 months (shorter than PFS in ZETA trial) in 60 locally advanced or metastatic MTC (91). This study confirmed the clinical benefit of vandetanib, reporting a PR in 20% of cases and SD in 55%.

Kim et al. (92) confirmed the results of the ZETA trial in terms of PFS and ORR, in a small cohort (n=12) of locally advanced or metastatic MTC patients. Koehler et al. (93) reached a median PFS of 17 months in 41 MTC patients with locally advanced and/or metastatic disease.

In our monocentric experience in Pisa (94), we evaluated 79 MTC patients with advanced disease treated with vandetanib. Patients were classified according to a short (< 1 year) or long-term (> 1 year) time treatment. Median PFS of the 79 patients was 47 months, longer than ZETA trial (30.5 months). Moreover, when considering only the 24 (30.4%) patients who experienced vandetanib treatment for ≥ 48 months, PFS was still longer (54.5 months). Also, in another real life setting in France (95), the authors found a median PFS of 22.7 months in 76 MTC patients treated with vandetanib, but it reached 73.2 months in the subgroup (n=21) who experienced treatment for more than 48 months, and in this subgroup the ORR was 87.5%. In younger patients and in those in whom vandetanib treatment was started without evidence of tumour progression, but because of severe disease related symptoms, clinical response was better and durable.

The results of this study were confirmed by Koehler at al (93). who found a longer PFS in patients ≤ 60 years at the beginning of vandetanib treatment and in patients with ≥ 5 adverse events.

The main limitation in the clinical use of vandetanib, like for other MKIs, is the presence of the off-target treatment emergent adverse events (TEAEs). The frequent onset of multiple TEAEs, some of them of grade ≥ 3 according to the Common Terminology Criteria for Adverse Events (CTCAE) (96), usually requires reduction or withdrawal of the drug, with unknown effects on its efficacy. The most frequent TEAEs, observed both in clinical trials (57) and in real life settings (92) are diarrhoea, nausea, cutaneous rash, and hypertension (**Table 2**). The management of TEAEs often requires a multidisciplinary approach by trained team and also patient training can be very helpful (97). If some TEAEs can be manageable with the use of specific drugs (i.e., hypertension), some other cannot and needs to be managed through drug reduction or withdrawal. A key role in the management of the TEAEs should be their prevention, but unfortunately no preventive and/or symptomatic treatments are available for each TEAEs, and in specific cases only supportive treatments could be available (i.e., fatigue) (97). Moreover, also in cases of additional outreach evaluating the TEAEs of the patients, no improvement on their rate and severity was demonstrated if compared to the standard monitoring schedule (98). Since the efficacy of the drug was demonstrated and TEAEs can be also correlated to the dose of the drug, a clinical trial (99) (ClinicalTrials.gov Identifier Nbib1496313) has been designed to compare the benefit–risk of two starting doses of vandetanib (300 vs 150 mg/daily).

**Table 2:** Most frequent treatment emergent adverse events (TEAEs) reported in the clinical trials and in the real-life studies.

	Clinical Trials (57–60)				Real-life studies (94, 95)	
	Vandetanib - Phase III (n=231)	Cabozantinib - Phase III (n=214)	Selpercatinib - Phase I/II (n= 143)	Pralsetinib - Phase I/II (n= 122)	Vandetanib - Valerio et al. (n=79)	Vandetanib - Ramos et al. (n=76)
Any grade (%)*	Diarrhea (56) Rash (45) Nausea (33) Hypertension (32) Headache (26) Fatigue (24) ↓Appetite (21) Acne (20) Dry skin (15) Acneiform dermatitis (15)	Diarrhea (63.1) Hand-foot syndrome (50) Weight loss (47.7) ↓Appetite (45.8) Nausea (43) Fatigue (40.7) Dysgeusia (34.1) Hair color changes (33.6) Hypertension (32.7) Stomatitis (29)	Dry mouth (39) Hypertension (30) ↑AST (28) ↑ALT (26) Fatigue (25) Oedema (18) Diarrhea (17) Constipation (16) Nausea (15) ↑Blood creatinine (14)	↑AST (33) Constipation (27) ↓WBC (26) ↑ALT (22) Hyperphosphataemia (22) Asthenia (22) Neutropenia (20) Anaemia (19) ↑Blood creatinine (18) Musculoskeletal pain (18)	Hypothyroidism (97.5) Rash (41.7) Diarrhea (30.4) Asthenia (30.4) Hypertension (26.6) Nausea (20.3) Biochemical alteration (19) Dysgeusia (11.4) Weight loss (8.9) Corneal alterations (6.3)	Folliculitis (73.7) Asthenia (54) Diarrhea (56.6) Hypertension (34.2) QT prolongation (30.2) Hypocalcemia (23.7) ↑AST/ALT (21) Anorexia (17) Weight loss (19.7) Nausea (9.2)
**Grade ≥ 3** (incidence ≥ 2%)	Diarrhea (11) Hypertension (9) QT prolongation (8) Fatigue (6) ↓appetite (4) Rash (4) Asthenia (3)	Diarrhea (15.9) Hand-foot syndrome (12.6) ↓weight (4.7) ↓appetite (4.7) Fatigue (9.3) Hypertension (8.4) Mucosal inflammation (3.3) Asthenia (5.6) Dysphagia (4.2)	Hypertension (12) ↑ALT (10) ↑AST (7) Diarrhea (3) QT prolongation (2)	Hypertension (17) Neutropenia (13) Lymphopenia (11) Anemia (10) ↓WBC (8) Asthenia (4) ↑CPK (4) Pneumonitis (3) Diarrhea (2)	Nausea (8.9) Rash (7.6) Hypertension (5) Asthenia (3.8) Diarrhea (2.5) Neuropathy (2.5) Pancreatitis (2.5)	QT prolongation (10.5) Diarrhea (5.2) Renal failure (3.9) Acute pancreatitis (3.9) Asthenia (10.5) Basocellular carcinoma (2.6) Pulmonary thromboembolism (2.6)
**Dose reduction rate**	35%	79%	30%	46%	–	36.8%
**Dose interruption rate**	–	65%	–	54%	–	39%
**Discontinuation rate for TEAEs**	12%	16%	2%	4%	17.7%	23.7%

AST, aspartate aminotransferase; ALT, alanine aminotransferase; WBC, white blood cells; CPK, creatine phosphokinase.

^*^For pralsetinib, since any grade TEAEs data were not reported, grade 1-2 TEAEs were shown.

Patients were randomized to receive vandetanib 150 or 300 mg/daily (1:1) for a maximum time of 14 months (Part A – n=81 pts). At the end of this first part, all patients had the possibility to enter in the open-label phase (Part B - n=61 pts) investigating vandetanib at various dosages (100, 150, 200, and 300 mg/daily). Significant ORR was experienced by 25% of the patients after 14 months of treatment both for patients treated with 300 mg (HR = 0.29; 95%CI: 0.176-0.445) and 150 mg (HR = 0.20; 95%CI: 0.105-0.348). In addition, in patients enrolled in Part B of the study, safety and tolerability were comparable to Part A. However, although not significant, a higher incidence of TEAEs was experienced by the patients treated with 300 mg compared with 150 mg. Therefore, the results of the trial confirmed that the most appropriate starting dose of vandetanib is 300 mg/daily and dose reductions can be considered to manage TEAEs. Conversely, lower starting doses should be considered in peculiar settings, such as patients with comorbidities.

Treatment with vandetanib was demonstrated to be safe and effective also in controlling MTC in children (100) and paraneoplastic Cushing’s syndrome due to ectopic ACTH secretion (101–104).

### Cabozantinib

Cabozantinib (XL184) is a MKI negatively influencing the activity of VEGFR-1 and VEGFR-2, MET, RET and c-KIT (105). FDA and EMA approved the use of cabozantinib after the publication of results of the double-blinded, phase III EXAM trial (ClinicalTrials.gov Identifier, NCT00704730). In the phase III EXAM trial (58), 330 patients with progressive advanced metastatic MTC were enrolled and randomized (2:1) to cabozantinib (140 mg/day) or placebo. PFS (11.2 vs 4.0 months; HR = 0.28; 95%CI: 0.19-0.40; p <0.001) and ORR (28 vs 0% - p <0.001) were significantly higher in cabozantinib, compared to placebo group.

In the same study population, a following analysis was performed assessing OS (70). Patients treated with cabozantinib showed a higher, but not significant, median OS than placebo group (26.6 vs 21.1 months). However, the analysis of the specific subgroup harbouring the *RET^M918T^* mutation emphasized the greater efficacy of cabozantinib compared to placebo (44.3 vs 18.9 months; HR = 0.60; 95%CI:0.38–0.94; p = 0.03). The authors cannot demonstrate the same result in *RET^M918T^* negative patients (20.2 months for cabozantinib versus 21.5 months for placebo group). An exploratory analysis of EXAM trial data evaluated the impact of *RET* and *RAS* mutations on PFS (106). PFS was significantly longer in cabozantinib group compared to placebo in patients harbouring *RET* mutations (60 vs 20 weeks; HR = 0.23; 95%CI: 0.14-0.38; p < 0.0001), mostly in *M918T*-mutant patients (61 vs 17 weeks; HR = 0.15; 95%CI: 0.08-0.28; p < 0.0001), in patients with unknown *RET* mutations (48 vs 13 weeks; HR = 0.30; 95%CI: 0.26-0.57; p =0.0001) and in those with *RAS* ones (47 vs 8 weeks; HR = 0.15; 95%CI: 0.02-1.10; p=0.0317). Conversely, in *RET* or *RAS* negative patients PFS did not differ between cabozantinib and placebo group. This clinical evidence emphasized the greater efficacy of cabozantinib treatment in patients with *RET^M918T^* patients.

TEAEs were very frequently experienced by patients treated with cabozantinib. Particularly, diarrhoea, palmar-plantar erythrodysesthesia, decreased weight and appetite, nausea, and fatigue (**Table 2**). Most patients experienced a dose reduction due to the TEAEs (79%) and some of them (16%) discontinued the treatment. Severe adverse events (SAEs), such as haemorrhages and intestinal perforation, were reported and correlated to the high antiangiogenic activity of cabozantinib against VEGFR (58).

### Vandetanib vs Cabozantinib: Is a Comparison Possible?

Currently, no one-to-one comparison between vandetanib and cabozantinib is available. Both these drugs are effective options in the treatment of advanced metastatic MTC. Vandetanib and cabozantinib treatments differed in median PFS reported in clinical trial and in clinical practice. The median PFS in the EXAM trial (11.2 months) was shorter than those reported in the ZETA trial (31 months) (57, 58, 107). However, these data are not fully comparable because of the difference in the inclusion criteria of the patients in the two trials as assessed by the different PFS of patients treated with placebo (19.3 vs 4 months, in ZETA compared to EXAM trial). Real-life data (93) showed that PFS in vandetanib (n=41) was higher than cabozantinib group (n=7) (17 vs 4 months), however the best response was PR in 12 (26%) patients treated with vandetanib and 5 (22%) treated with cabozantinib. These data are probably affected by small sample size and the difference in the number of patients treated with vandetanib compared to cabozantinib, making very difficult the comparison of the results.

Although a one-to-one comparison is not possible, each drug shows peculiarities that can influence the choice of one or another drug in the clinical practice. First, both drugs are not always available and refundable in all countries, and this necessarily influences the choice. Thus, whenever both drugs are available, the safety profile of the drugs becomes preponderant. Some TEAEs are commonly experienced with both drugs, such as diarrhoea, nausea, fatigue (57, 58, 94, 95) and alteration of thyroid function (108), however the safety profile of the 2 drugs is slightly different. Vandetanib is contraindicated in patients who have a prolonged QTc (> 450 ms in men and > 470 ms in females) (109), while cabozantinib in patients with a history or presence of diverticulitis (58). Proteinuria is a late-onset adverse event in patients treated with cabozantinib (110). Moreover, cabozantinib demonstrated its efficacy also as a second-line treatment (107) while this information is not available for vandetanib.

During the treatment with both drugs, sooner or later, resistance appeared. *In vitro* studies described that the *V804L/M RET* mutation conferred a primary resistance to vandetanib (111). The presence of this mutation seems to not limit the efficacy of cabozantinib and for this reason in patients harbouring *V804L/M* mutation, cabozantinib should be preferred.

Moreover, secondary resistance mechanisms due to the development of several different *RET* mutations using RET kinase-dependent *BaF3/KIF5B-RET* cells (i.e., *L730I, E732K, Y806N, G810S, V871I, G810S/G949R*), were associated with vandetanib and cabozantinib treatment (85).

## Other MKIs Tested in the Treatment of Advanced MTC

Over the years, several MKIs were tested for the treatment of advanced MTC although they never reached the clinical practice. Here we reported the available data about the phase II trials that enrolled more than 15 patients.

### Motesanib

The first MKI tested in a phase II trial was motesanib, inhibitor of VEGFR-1, 2 and 3, platelet-derived growth factor receptor (PDGFR), stem cell factor receptor (c-Kit) and RET (112, 113). The results of phase II trial evaluating the safety and efficacy of motesanib in MTC (ClinicalTrials.gov Identifier NCT00121628) were published in 2009 (77). Ninety-one patients with locally advanced or metastatic, progressive, or symptomatic MTC, were enrolled to receive motesanib 125 mg/daily. Partial response was obtained in 2% of the patients, while 81% of cases showed SD. Median PFS was 48 weeks (95% CI, 43 to 56 weeks). TEAEs of any grade were experienced by 88% of the patients but about 40% were of grade ≥3 or above of which the most common were diarrhea, fatigue, and hypertension.

### Sorafenib

Sorafenib (BAY 43-9006) is a MKI of Raf-1 and B-Raf, VEGFR-2 and 3, PDGFR-β, Flt-3 and c-KIT (114). The safety and efficacy of sorafenib in treating metastatic MTC patients was tested in a phase II trial (ClinicalTrials.gov Identifier NCT00390325) (78). The patients were treated with 400 mg/twice daily and were divided in arm A (n=5) with hereditary MTC and arm B (n=16). In arm A, 1 patient had PR, and the remaining 4 patients showed SD, but arm A was prematurely closed for the difficulties in recruiting patients with metastatic progressive hereditary MTC. In arm B, 1 patient was not evaluable, 1 achieved PR (6%; 95%CI, 0.2-30.2%), and the remaining 14 had SD (88%; 95%CI, 61.7-99.5%). The most common TEAEs of grade ≥ 3 grade were diarrhea, hand-foot-skin reaction, and hypertension.

### Pazopanib

Pazopanib, a MKI of VEGFR1, 2 and 3, PDGF, c-Kit, fibroblast growth factor receptor (FGFR) 1, 3 and 4 and RET (115) was tested in a phase II trial for advanced, progressive MTC (ClinicalTrials.gov Identifier NCT00625846) (79). Thirty-five patients with advanced MTC showed progression in the 6 months before the enrollment, received pazopanib (800 mg/daily) until disease progression or unacceptable toxicity. Among patients treated with pazopanib as first line therapy (n=20), 3 showed PR (response rate 15%; 95%IC: 4.2%–34.4%), 11 had SD, 4 progressed before the second evaluation and 2 discontinued the treatment for reasons other than progression. Median PFS was 9.4 months, and OS was 19.9 months. The two most frequent ≥ 3 Grade TEAEs were diarrhea and fatigue.

### Lenvatinib

Lenvatinib is a MKI of the VEGFR-1, 2 and 3, FGFR 1–4, PDGFR alpha, RET, and proto-oncogene c-KIT (**Table 1**). The efficacy of Lenvatinib (24 mg/daily) in the treatment of MTC was evaluated in a phase II trial (116) (ClinicalTrials.gov Identifier NCT00784303), in which 59 patients with advanced, unresectable MTC which showed progression of the disease according to RECIST, were enrolled. ORR was 36% without difference between patients who previously received other anti-VEGF therapy (n=26; 44%) or not, and the median PFS was 9 months. The most frequent grade 3-4 TEAEs were diarrhoea, hypertension, asthenia, loss of appetite and dysphagia. Despite the promising results, lenvatinib was never approved for treatment of the MTC but only for the treatment of locally recurrent or metastatic, progressive, radioactive iodine-refractory differentiated thyroid cancer (68).

However, according to the promising results of phase II trial, we recently reported data regarding the “off label” use of lenvatinib as salvage therapy in a cohort of patients with advanced and progressive MTC previously treated with other MKIs, discontinued for disease progression or severe TEAEs (117). We observed an early and quite durable stabilization of the disease, particularly in the cervical lymph nodes. Conversely, the efficacy on bone metastases was demonstrated in approximately 60% of cases.

Toxicity profile of lenvatinib was characterized by predominantly grade 2 or lower TEAEs.

### Anlotinib

Anlotinib is a MKI of VEGFR, FGFR, PDGFR, and c-Kit. According to its mechanism of action it was able to reduce both tumour angiogenesis and cell proliferation (86, 118, 119). According to the promising results of preclinical data (86) and phase I trial (120), phase II trials were designed. The first phase II trial (121) showed high activity against MTC, in 58 patients with unresectable or metastatic naïve MTC (without any previous treatment with anti-angiogenic agents), reaching an ORR of 56.9% and a PFS rate of 92.2%, 87.8% and 84.5% at 24, 36 and 48 weeks, respectively. Recently, the results of the multicenter, randomized, double-blind, phase IIB clinical trial (ALTER 01031) (ClinicalTrials.gov Identifier NCT02586350) have been published (122). Ninety-one patients were enrolled and randomized to receive anlotinib (12 mg once daily from day 1 to 14 every 3 weeks) or placebo in a 2:1 ratio. The trial evaluated the efficacy (PFS and ORR) and toxicity profile of anlotinib. Median PFS was significantly higher (20.7 vs 11.1 months; HR = 0.53; 95%CI: 0.30–0.95; p = 0.029) in the anlotinib (n=62) than in placebo group (n=29). The ORR of anlotinib group was 48.4%. The most common TEAEs of anlotinib were palmar–plantar erythrodysesthesia syndrome, proteinuria, and hypertriglyceridemia (**Table 2**).

## Ret Selective Inhibitors

Varying from 43 to 71% of sporadic MTC harbor somatic *RET* mutations (38, 123, 124). However, when considering only advanced metastatic cases, the prevalence of *RET* mutations, particularly *M918T*, significantly increased up to 85% (125). *RET*-mutant MTC showed aggressive clinical behavior compared with MTC carrying other mutations, due to the higher risk of lymph nodes, distant metastases, and worse survival (75, 123, 126).

Aberrant RET signaling, like other oncogenes, can enhance the proliferative signaling, particularly the cell proliferation, an essential mechanism to sustain the tumor growth (127–129). Indeed, RET has been considered a driver gene in other thyroid tumors such as papillary thyroid carcinoma, but also in lung, breast and colon cancers (130).

Interestingly, aberrant RET signaling is not a driver event in all cell types, being involved in several additional mechanisms in the process of tumor genesis (131). RET signaling can be involved in the evasion of growth suppression, resistance to the cell death, progress of the replicative immortality (132), induction of the angiogenesis (56) and activation of the tumor invasion and metastasization (132–134). Thus, RET represents an ideal actionable oncoprotein and its highly selective inhibition could be effective in the treatment of many cancers.

Although their efficacy against the tumor and despite their activity against RET, the first-generation MKIs approved for the treatment of MTC (i.e., vandetanib and cabozantinib) were limited in their use by the onset of the off-target toxicities (40, 100, 110, 135). Therefore, a second-generation of highly selective *RET* inhibitors were developed both to maintain the anti-tumor efficacy and to improve the safety profile.

Selpercatinib (LOXO-292) and pralsetinib (Blu-667) (136, 137) showed potent inhibition of RET, when compared with MKIs, had high bioavailability and significant central nervous system penetration (136). Although they are highly selective for RET, they also show a low potency, much lower than the other MKIs, on VEGFR2 (**Table 1**) (138).

They can inhibit the proliferation of cells harboring several types of *RET* mutations, both gene fusions and point mutations, including the gatekeeper *V804M/L* mutation which, as previously reported, showed *in-vitro* resistance to vandetanib (111). Efficacy of selpercatinib and pralsetinib in terms of tumor shrinkage was confirmed in animal models and clinical studies (136, 137). Moreover, the activity of selpercatinib in treating brain metastases was highlighted in mouse models (136).

Two phase I/II studies were built to evaluate the efficacy and safety of selpercatinib (59) and pralsetinib (60) in treating patients with *RET-*mutant thyroid cancers. Main features of these studies and the comparison with the ZETA and EXAM trials are reported in **Table 3**.

**Table 3:** Efficacy data of clinical trials evaluating vandetanib, cabozantinib, selpercatinib and pralsetinib treatments in MTC patients.

		Vandetanib (57)	Cabozantinib (58)	Selpercatinib (59)	Pralsetinib (60)
Trial design		Double-blind, randomized, placebo-controlled	Double-blind, randomized, placebo-controlled	Open label	Open label
Clinical phase		III	III	I (dose escalation) II (dose expansion)	I (dose escalation) II (dose expansion)
N° of patients	Treatment	231	219	143	122
	Placebo	100	111	–	–
Initial drug dose		300 mg	140 mg	Phase I: from 20 mg QD to 240 mg BID Phase II: 160 mg BID	Phase I: from 30 mg QD to 600 mg QD Phase II: 400 mg QD
MKIs naïve patients		90/231(63.8%)	44/219 (20%)	55/143 (38.4%)	23/84* (27.3%)
ECOG performance status	0	154 (66.7%)	123 (56.2%)	54 (37.7%)	32 (38.1%)
	1	67 (29.0%)	95 (43.4%)	83 (58.0%)	49 (58.3%)
	2	10 (4.3%)		6 (4.2%)	3 (3.6%)
Main outcome		PFS	PFS	ORR	ORR and safety
Secondary outcome		ORR, DCR, OS, biochemical response, time to worsening pain	OS and ORR	DOR, PFS, and safety	DOR, CBR, DCR, PFS and OS
PFS		30.5 months (treatment) 19.3 months (placebo)	11.2 months (treatment) 4.0 months (placebo)	23.6 months (MKIs naïve) 27.4 months (pre-treated)	Not reached
ORR		45%	28%	70% (MKIs naïve) 61% (pre-treated)	72% (MKIs naïve) 60% (pre-treated)
DCR		87%	76%	87% (MKIs naïve) 84% (pre-treated)	100% (MKIs naïve) 93% (pre-treated)

ECOG, Eastern Cooperative Oncology Group; PFS, progression-free survival; ORR, objective response rate (complete + partial response according to RECIST 1.1); MTD, maximum tolerated dose; DOR, duration of response; DCR, disease control rate; OS, overall survival; CBR, clinical benefit rate.

^*^This number is referred to evaluated patients only.

**Figure 2:**
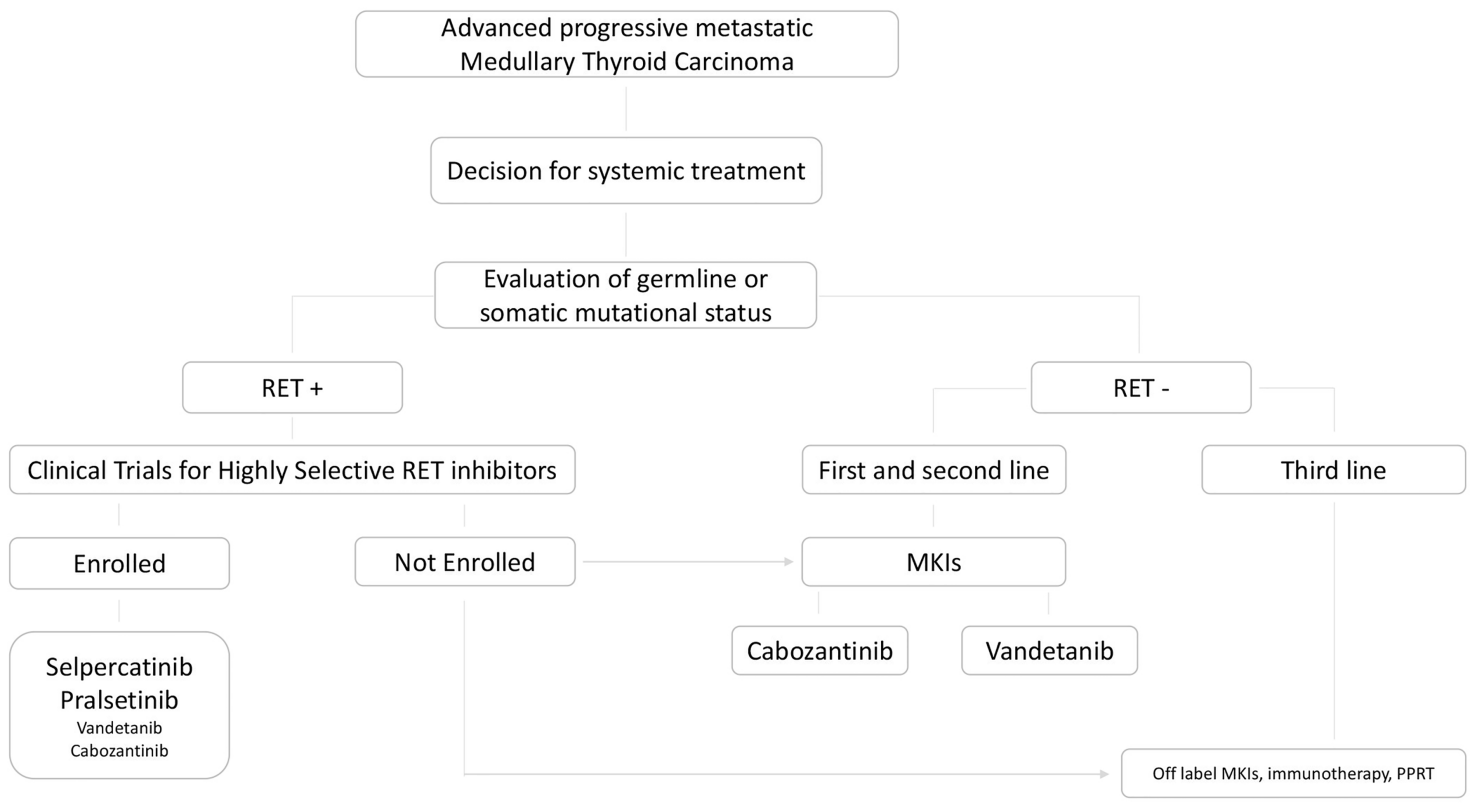
State of art of therapeutic choice in advanced, progressive, metastatic MTC, according to the presence or absence of RET germline or somatic mutation.

Moreover, in **Figure 2** we highlighted the key points in the treatment of advanced metastatic progressive MTC according to the RET mutational status.

### Selpercatinib

LIBRETTO-001 clinical trial (ClinicalTrials.gov, Identifier NCT03157128) (59) enrolled 531 patients of whom 162 with RET-mutant thyroid cancers, between May 2017 and June 2019. The study integrated a phase I in which the patients received selpercatinib with different starting doses (from 20 mg once daily to 240 mg twice daily) and experienced progressive decreases up to reach the highest safe dose (escalation dose), and a phase II in which all patients started with the same dose of 160 mg twice daily.

*RET* alteration status was evaluated in local certified laboratories using several techniques [next-generation sequencing (NGS), fluorescence *in situ* hybridization (FISH), or polymerase-chain-reaction assay (PCR)]. Central confirmation was not required.

*M918T* was the most frequent *RET* mutation found (about 60%) in both groups of MTC, while cysteine rich domain *RET* mutations were present in about 15% of the cases. In 7-9% of the cases also *V804 M/L* gatekeeper mutations were present.

The study aimed to assess the ORR (complete or partial response according to RECIST), the PFS, the DOR and the safety of the treatment. In this study, radiological progression of the disease according to RECIST within 14 months from the screening visit was an inclusion criteria for *RET*-mutant MTC patients but not for *RET*-fusion positive thyroid cancer.

Most of patients (143/162 – 88.3%) had MTC, 55 (38.5%) already treated with vandetanib and/or cabozantinb and 88 (61.5%) without any previous MKIs treatment (naïve). The remaining patients (19/162 – 11.7%) showed a *RET* fusion–positive thyroid cancer (PTC, poorly differentiated thyroid carcinoma, Hürthle cell carcinoma and ATC). Radiological assessment was performed both by an independent review and by the investigators. According to independent review, in patients already treated with vandetanib and/or cabozantinib, ORR rate was 69%, while in naïve patients was 73%. ORR rate was similar (79%) also in the remaining patients with *RET* fusion-positive thyroid cancers.

Also, tumor markers, CTN and CEA, showed an outstanding response to the treatment decreasing up to 100% in several cases.

These results were confirmed regardless of *RET* mutations considered, including the *V804M/L*. After a median time of 1 year, 82% of MTC patients previously treated with vandetanib and/or cabozantinib, 92% of the naïve and 64% of other *RET*-mutant thyroid cancers remained free of progression. However, duration of response was not achieved, and longer follow-up data are needed.

TEAEs of any grade, regardless of attribution to the drug, were experienced by 94% of patients, and most frequent were: dry mouth (46%), hypertension (43%), diarrhea (38%), fatigue (38%), increased aspartate aminotransferase levels (35%) or alanine aminotransferase levels (31%) and constipation (35%). TEAEs attributable to the drug are mainly of grade 1 and 2 according to CTCAE and dry mouth (39%) and hypertension (30%) were the most frequent. However, the prevalence of TEAEs for single patient was lower and most of them were of lesser intensity, compared with TEAEs experienced with vandetanib (57) and cabozantinib (58) (**Table 2**). The safety profile was the same across all cancers treated in LIBRETTO-001 (59) and if considering all patients enrolled, including those with lung cancer, only 30% of them experienced a dose reduction for TEAEs and only 12 on 531 patients (2%) a discontinuation of the treatment (59, 139).

Efficacy of selpercatinib treatment in *RET*-mutant MTC patients was highlighted also in several anecdotal cases. After treatment with selpercatinib, complete and durable response of the measurable brain and leptomeningeal metastases was demonstrated in a *M918T RET*-mutant MTC patient who experienced previous disease progression on cabozantinib and radiation therapy (140). Also, in a rare, and probably underestimated setting, a *M918T RET*-mutant MTC patients who experienced complete visual loss due to the presence of choroidal metastases, the third line treatment with selpercanib, after progression of the disease on vandetanib and off-label lenvatinib, determined the disappearance of the choroidal metastases and sight rescue (141). Moreover, clinical utility of selpercatinib was showed also in a neoadjuvant setting, determining a RECIST response greater than 50% followed by complete surgical resection in a *RET*-mutated MTC patient with initially unresectable, widely metastatic disease (142).

Lastly, in peculiar setting of pediatric patients, selpercatinib was useful to control the disease, in absence of relevant TEAEs, in 2 patients with *RET*-mutant MTC who experienced loss of clinical benefit with previous MKIs treatment (143).

Based on these results, selpercatinib has been approved by the FDA and EMA for adult and pediatric patients (≥ 12 years) with advanced or metastatic RET-mutant MTC who had previously been treated with cabozantinib, vandetanib or both.

A multicenter, randomized, open-label, phase III trial comparing selpercatinib to physicians’ choice of cabozantinib or vandetanib (standard of care) in patients with progressive, advanced, kinase inhibitor naïve, *RET*-mutant MTC (LIBRETTO-531) is ongoing and recruiting patients (ClinicalTrials.gov Identifier: NCT04211337).

### Pralsetinib

The ARROW trial integrated a phase I dose escalation (from 30 to 600 mg once daily) to establish the maximum tolerated dose of pralsetinib, and phase II expansion cohorts (400 mg/daily) enrolling patients with *RET*-mutant cancers (ClinicalTrials.gov, Identifier NCT03037385) (60).

From March 2017 up to July 2020, 521 patients with *RET*-mutant cancers were enrolled. Of these, 147 had *RET*-mutant MTC, and 22 had *RET* fusion positive thyroid cancers. In the phase II, 122 MTC patients and 20 *RET* fusion positive thyroid cancer patients were included in the safety analysis. Sixty-one MTC patients who experienced previous treatment with cabozantinib and/or vandetanib, 23 naïve MTC patients and 11 patients with *RET* fusion positive thyroid cancer were included in the efficacy analysis.

To be included in the study, *RET*-mutant MTC patients had to show progression, according to RECIST 1.1 within 14 months before the screening visit. For *RET* fusion positive thyroid cancer, progression of the disease was not required.

*RET* alterations were assessed by several local testing methods, NGS using DNA or RNA, in tumor tissue or in circulating blood, or FISH in tumor tissue.

The main outcome of phase 2 study was the ORR. Other endpoints were PFS, DOR, clinical benefit rate (rate of patients with stable disease or partial or complete response for more than 16 weeks), disease control rate (rate of patients with stable disease or partial or complete response) and OS. For MTC also values of CTN and CEA were collected.

Of the 61 patients with *RET*-mutant MTC previously treated with MKIs, 41 (67%) had *M918T* mutation, 14 (23%) had mutation in the cysteine rich domain, 2 (3%) had *V804M/L* mutations, and 4 (7%) had other *RET* mutations. Conversely the most frequent mutations detected in *RET*-mutant naïve MTC were those involving the cysteine rich domain (n=12 – 52%).

ORR was 60% in the MTC patients who experienced previous treatment with MKIs, while in the naïve MTC group ORR was 71%. Two cases, 1 in pre-treated and 1 in the naïve group, showed a complete response to the treatment. The results were independent of *RET* mutations considered, including the *V804M/L*. In *RET* fusion positive thyroid cancer group ORR was 89%.

Biochemical markers of MTC, CTN and CEA, significantly decreased in most of patients.

The most frequent TEAEs of any grade (**Table 2**) were anemia (45%), musculoskeletal pain (45%), constipation (44%), increased aspartate aminotransferase (42%) and hypertension (40%). SAEs were reported in 15% of the patients and the most frequent was pneumonia (4%). Dose was decreased in in 44% of the patients mainly due to anemia, lymphopenia or neutropenia and hypertension and 54% of the patients experienced a dose interruption for TEAEs. However, only 5 patients permanently discontinued the treatment.

Based on these results, pralsetinib was approved by the FDA for treatment of advanced or metastatic RET-mutant MTC.

A phase III, randomized, open-label trial of pralsetinib versus standard of care (vandetanib or cabozantinib) for treatment of *RET*-mutated MTC (AcceleRET-MTC) has been designed and the scheduled start date is January 2022 (ClinicalTrials.gov Identifier: NCT04760288).

### Mechanisms of Resistance to Selective RET Inhibitors

As it happens with MKIs, despite the very interesting efficacy and safety profile of highly selective RET inhibitors, acquired resistance can be developed. Despite their efficacy on several *RET* mutations, including the gatekeeper *V804M/L*, there are emerging data about resistance mechanisms involving non gatekeeper mutations.

In one NSCLC patient, harboring *KIF5B-RET* rearrangement, circulating tumor DNA and post-mortem biopsy analysis showed the appearance of a wide spectrum of *RET* mutations on *G810* residue (solvent front mutations), concomitantly with progression of the disease. The appearance of these mutations was related to tumor progression and confirmed in other progressing NSCLC and MTC cases, and *in-vivo* and *-vitro* models (144). Selpercatinib and pralsetinib showed a peculiar mechanism of wrapping around the tyrosine kinase, being able to escape the resistance caused by the gatekeeper mutations V804, but unfortunately susceptible to other RET mutations such as *V738A, Y806C/N* and *G810C/S* (145). These mutations are clinically relevant and can play a key role in the resistance mechanisms, although occurring at relatively low frequency (131, 144).

Most of the resistance to highly selective RET inhibitors derived by RET-independent mechanisms, such as *MET* and *KRAS* amplification, and were described in progressing NSCLC patients (146, 147). In patients with *MET* amplifications, no additional concomitant *RET* mutations were described (146) and the association of MET inhibitor, such as crizotinib, to the RET inhibitor, was able to overcome the resistance, showing a clinical benefit (147–150).

Another mechanism of resistance recently discovered was the novel appearance of *NTRK3* fusion in a patient with *RET* fusion positive high-grade neuroendocrine carcinoma who progressed after an initial response to the selpercatinib treatment (151). Also, in this case, the resistance to selpercatinib could be potentially overcome by adding an NTRK inhibitor such as entrectinib (152) or larotrectinib (153).

#### Future Directions

Since resistance mechanisms were highlighted both with MKIs and with second generation highly selective RET inhibitors, a third generation of RET inhibitors are being investigated to target the additional *RET* mutations responsible for resistance to the previous drugs. TPX-0046 is a RET/SRC inhibitor designed to occupy less space in the RET-binding pocket, restricting the area of the kinase domain that can mutate and cause resistance, maintaining strong antitumor activity. Moreover, by inhibiting *SRC*, this drug can block SRC-driven resistance observed during the treatment with RET inhibitors (154). TPX-0046 demonstrated a higher inhibition when compared with selpercatinib and pralsetinib, against the *RET* solvent front mutations (155). In December 2019, a phase I/II open-label trial to determine the safety, tolerability, pharmacokinetic, and preliminary efficacy of TPX-0046 in adult subjects with advanced or metastatic solid tumors harbouring *RET* mutations or alterations (ClinicalTrials.gov Identifier: NCT04161391), started. The study is active and recruiting and estimated primary completion date is May 2024.

BOS-172738 is another small-molecule, showing *in vitro* and *in vivo* RET inhibition. It was tested in a phase I trial in patients with advanced solid tumours with *RET* gene alterations including NSCLC and MTC (ClinicalTrials.gov Identifier: NCT03780517) (156). Recently, preliminary data were reported about 16 patients with *RET*-mutant MTC enrolled in the trial showing an investigator-assessed ORR of 44% (7/16 patients with 1 complete response). Safety profile was interesting with most TEAEs classified as grade ≤ 2 and considered not related to the drug; the most common were creatinine phosphokinase (CPK) increase (54%), dyspnoea (34%), facial oedema, aspartate aminotransferase elevation, anaemia (25% each), neutropenia, diarrhoea (22% each), fatigue (21%), and constipation (20%) (157). The trial is active, but not more recruiting, 117 patients have been enrolled and the estimated primary completion date is December 2021.

Another selective RET inhibitor TAS0953/HM06 is being tested in a phase I/II trial in patients with advanced solid tumours with *RET* gene abnormalities: the trial is open and recruiting subjects (ClinicalTrials.gov Identifier: NCT04683250). Other compounds such as SL-1001 showed promising activity against *RET*-driven tumor models (158). The development of selective RET inhibitors against specific mutations may provide a key alternative option to patients that develops resistance and clinically progressed over time during treatment with highly selective RET inhibitors.

## Testing for Ret Mutation: Who and When

Clinical management of patients with MTC favorably changed over time after the introduction of genetic screening for *RET* germline mutation in clinical practice. About 25% of all MTCs are inherited through an autosomal dominant trait (159). Moreover, about 6% of apparently sporadic MTC, are hereditary after performing *RET* genetic screening (9). Therefore, according to the main guidelines (1, 159), genetic screening for *RET* germline mutation should be performed in all patients with MTC. In the remaining 75% of cases, MTC is sporadic. In these cases, somatic *RET* mutations are detected in about 25%–40% of the cases (160, 161). The most common *RET* somatic mutation occurred in codon *M918* within exon 16 and is detected in more than 85% of *RET*-positive cases, followed by *C634* within exon 11 (71, 74). The presence of *RET* somatic mutations in sporadic MTC have a recognized negative prognostic value (38).

Thus, in case of negative *RET* germline mutation, the question of whether to search or not for somatic mutations and when, is a crucial topic.

Currently, there is no indication in the ATA guidelines (1) for when and if to perform somatic mutations when available. Conversely, in the last update of NCCN guidelines (162), the genomic testing, including tumor mutational burden or RET somatic genotyping was suggested in case of symptomatic, progressive, metastatic disease, according to RECIST, in patients who are germline wild-type or germline unknown, before starting treatment with highly selective RET inhibitors.

However, since the new highly selective drugs are targeted against *RET* mutations, knowing the mutational status of sporadic MTC could become essential.

It is worth noting that only a subgroup of patients, those with advanced metastatic progressive MTC, will be treated with systemic therapies. Most of these patients are easily identifiable, already at the time of diagnosis, because of the presence of distant metastases. In these cases, knowing the mutational status of the primary tumor and, if possible, also of the local or distant metastases can be useful to plan the most appropriate therapeutic drug sequence. This is particularly recommended in cases that are supposed to be treated with systemic therapy in the next future. In all other sporadic MTC patients, both those who are cured after surgery and those with low probability of developing advanced disease, beyond the scientific purposes, the routine test for finding mutational status is not clinically advised.

Several techniques can be performed for the detection of *RET* alterations (163). The “nucleic acid-based assays”, particularly the next generation sequencing (NGS) should be the preferred one, followed by the real time PCR-based assay. The “*in situ* assays”, mainly immunohistochemistry (IHC) is rarely used because the lack of specific antibodies against *RET* mutation. Several reasons can guide the choice, mainly the accessibility to perform a specific technique rather than another. Moreover, the quantity and quality of tumoral tissue available is a key limiting factor for the molecular analysis, particularly in cases where the analysis is performed on the primary tumor tissue several years after surgery. Although the paraffin embedded tissue can be used to extract both DNA and RNA the quality of the nucleic acid is sometimes suboptimal to obtain reliable results.

It is worth noting that for some patients, mutation testing of the primary tumor or metastatic lesion is not feasible due to the lack of tissue or the low quality of DNA if tissue is available. In these cases, the liquid biopsy that is the search for mutations in circulating plasma cell-free DNA (cfDNA), could be performed as a surrogate analysis. However, if a somatic driver RET mutation is detected, we can use this information in the therapeutic algorithm; a negative result is not necessarily informative of a RET negative status, in absence of tumor tissue data (164).

The choice of the technique to perform can also regard the type (mutations or fusions) and the number of alterations to check, the context (oncogenetics or theranostics) and the cost. It is worth noting that, except for anecdotal cases, no RET fusions have been reported in MTC and no *RET* point mutation in DTC (71).

## Beyond MKIs and Highly Selective Inhibitors

### Targeting Other Systems/Pathways Involved in C-Cells Tumorigenesis

The pivotal role played by RET in MTC oncogenesis represents the key target for the rationale of MKIs and highly selective inhibitors treatment. However, several other leading actors can play a role in and around MTC cancer cells (165). RET downstream pathways (e.g., RAS/RAF/MAPK and PI3K/AKT/mTOR), other intracellular pathways (e.g., NOTCH), other main intracellular mechanisms (e.g., proteasome), other cell membrane receptors (e.g., somatostatin receptors – SSTRs) and immune response regulation, could influence MTC oncogenesis and could be a potential target of specific treatments.

RAS proteins family is one of the earliest described oncogenes, since they were characterized in mouse and man about 60 and 40 years ago, respectively (166, 167). This family include H-, K- and N-RAS, which regulate cell proliferation, metabolism and migration in physiological and pathological conditions (168). *RAS* mutations are commonly detected in many cancers, and they account for about 30% of somatic mutations detected in a large series of MTC (126). Although considered interesting targets for selective therapies, they had been recently defined “undruggable” (169). Currently, many strategies are developing to inhibit, directly or indirectly, RAS or their downstream partners (170). Tipifarnib inhibits a post-translational modification, namely farnesylation of RAS proteins. Farnesylation is a key modification for H-RAS protein, and its inhibition indirectly affect H-RAS function (171). In 2008, Hong et al. reported the case of a 37-years-old man affected by MTC, who after several surgical treatments, started tipifarnib alone for 4 weeks and in combination with sorafenib, after progression of the disease. At the end of follow-up (8 months), a partial response was achieved without any significant TEAEs (172). Following this interesting report, 13 patients with metastatic MTC were enrolled in a phase I trial exploring the efficacy and safety profile of the combined therapy tipifarnib and sorafenib (173). Most of the enrolled patients (11 out of 13) experienced PD before the enrollment. Median PFS was 15 months and best overall response was available in 10 patients who showed PR (50%) and SD (50%).

PI3K/AKT/mTOR is another RET downstream pathway (174), main regulator of cell growth, motility, survival, metabolism, and angiogenesis (175). In MTC samples, Tamburrino et al. showed that PI3K/AKT/mTOR is frequently activated: 49 and 40 out of 51 samples showed activation of mTOR and AKT, respectively (176). Intriguingly, mTOR activation was also present in 70% of cases of C-cell hyperplasia, conceivably indicating an early step for C-cell transformation (176). Accordingly, *in-vitro* models showed that the inhibition of the mTOR through a specific drug (everolimus) lead to a significant inhibition of cell growth, without affecting apoptosis (177) and in mouse model everolimus blocked tumor growth (176). These findings lead to test everolimus in clinical settings. A preliminary report showed its efficacy in a woman with progressive metastatic MTC, who experienced SD after more than 18 months of treatment (178). Likewise, Schneider et al. evaluated treatment with everolimus in 7 patients with progressive metastatic MTC, obtaining SD in 5 and PD in 2 patients (179). Hanna et al. obtained similar results in 10 patients with locally advanced or metastatic MTC treated with everolimus (8 SD, 1 PR and 1 PD) (180). However, disease progression was not required as inclusion criteria thus weakening the clinical meaning of disease stabilization observed. A recent report argued that a combination therapy of everolimus and pasireotide (SSTRs agonist) could produce some clinical benefit in progressive MTC (181), according to the expression of SSTRs in MTC cells (SSTR1, SSTR2, and SSTR5) (182).

NOTCH pathway is a cell-to-cell communication between a signal-sending and a signal-receiving cell (183). It can regulate angiogenesis, cancer stem cell fate, the immune response and resistance to therapy, both chemotherapy and targeted therapy (183, 184). Interestingly, NOTCH pathway was demonstrated to be inactive in MTC cell lines and its activation had inhibitory effects on tumor growth (185, 186). Accordingly, Delta-like ligand 3 (DLL3), an inhibitory NOTCH ligand, was upregulated in high grade neuroendocrine tumors (187) and in MTC, in which it correlates with lymph-node metastasis (188). Recently, a phase I/II trial showed that a DLL3-targeting antibody-drug conjugate (rovalpituzumab) could have antitumor activity in patients with MTC (ClinicalTrials.gov, Identifier NCT02709889) (189).

Protein catabolism system has a central role both in normal and in cancer cells and its correct function is critical for cell survival and proliferation (190). For this reason, it was studied as a target in cancer treatment (191) and bortezomib, a proteasome inhibitor, was proposed in MTC therapy. Bortezomib showed IC_50_ for MTC cells within the range of clinically achievable concentrations (192). In *in-vitro* model, bortezomib was able to inhibit NF-kB activity inducing expression of pro-apoptotic proteins such as p53 and promoting caspase-dependent apoptosis (192). However, its use in a phase I trial in combination with vandetanib did not achieve the expected results. RECIST response was available for 18 patients with metastatic or advanced MTC and the combination therapy with vandetanib and bortezomib, did not show any improvement if compared to vandetanib alone (ClinicalTrials.gov, Identifier NCT00923247) (193).

### Radiometabolic Treatment in MTC

The presence of SSTRs on MTC cells is the rationale for the use of peptide receptor radionuclide therapy (PRRT) with radiolabeled somatostatin analogs [90 yttrium (90Y), 177 lutetium (177Lu) and 111 indium (111In)]. After some pilot study demonstrating the clinical benefit of [90Y-DOTA]-TOC treatment (194, 195), a phase II trial was performed in metastatic MTC to evaluate safety and overall survival (196). Moreover, in the same study also the response to the treatment, defined as post-therapeutic prolongation of CTN doubling time of at least 100% was evaluated. A median cumulative activity of 12.6 GBq was administered in 31 patients. Response to the treatment was demonstrated in 29% of patients (9/31) and was associated with longer survival. Interesting results were reported also by Vaisman et al. who evaluated the efficacy of 177Lu-DOTATATE treatment in a phase IV trial enrolling patients who showed a positive uptake of the lesions at the 111In-DTPA-octreotide scan (ClinicalTrials.gov, Identifier NCT01915485) (197). Accordingly, 7 patients were treated and 3 of them achieved PR, 3 SD and only one patient a PD.

Although biochemical response was usually achieved, different results were experienced about structural response, and SD was the most frequent clinical response obtained. Furthermore, in a different cohort of 42 patients, almost 40% of them showed PD (198). Recently, a meta-analysis reviewed the efficacy of PRRT in patients with MTC, evaluating data from 220 patients (199). Unfortunately, the type of PRRT used was not described in 51 patients, most of the other patients were treated with 177Lu-based agent (n=157) and only a minority with 111In-based agent (n=12). Biochemical and structural response data were available in 145 and 134 patients, respectively. Although 54 patients had a decrease in CTN and/or CEA, only about 10% of patients experienced CR or PR. These data could suggest that only a small subset of patient could benefit from this treatment. Accordingly, Hayes et al. showed a low prevalence of high tumor uptake in metastatic MTC patients, performing 68Ga-DOTA-SSA PET/CT, and they argued that only in these highly selected patients PRRT may be an available and effective option (200).

Radioiodine treatment with ^131^I has a significant therapeutic role in differentiated thyroid cancer patients but not in MTC. MTC cancer cells are not sensitive to ^131^I because of the lack of sodium iodide symporter (201). Nevertheless, in the past, some author reported interesting data about the efficacy of radioiodine in *in-vitro*, animal models (202, 203) and in a single-report on 7 MTC patients (204). It was hypothesized that residual follicular cells, which were close to malignant c-cells, could entrap enough ^131^I to produce a bystander effect and destroy the c-cells (205). However, less than 10 years ago, a retrospective multicenter study showed that radioiodine treatment did not produce any effect on disease-free and disease-specific survival in MTC patients, confirming that ^131^I has no role in the treatment of MTC (206).

### Immunotherapy in MTC

Regulation of immune system made by cancer cells is a well-established hallmark of cancer (207). Since the jailbreak of the immune response is frequent across different cancers, it is not surprising that the re-activation of immune reaction toward cancer cells have recently become a relevant therapeutic weapon against several cancers (208). However, the impact of immunotherapy in MTC patients was less significant than other cancer, although pioneering studies reported intriguing results (209). Indeed, more than 40 years ago, Rocklin et al. described the immune response induced by MTC tumor lysate (210).

More recently, some evidence argued that MTC tumor microenvironment could be immunologically “colder” than imagined in the past. Bongiovanni et al. evaluated the expression of PD-L1 in a small series of MTC (211). Their results were disappointing: in 16 samples, only in one case PD-L1 expression was pointed out. However, 22 and 19 MTC samples showed an expression for PD-1 and PD-L1, in a quite large cohort of Chinese patients with MTC (n=87) (212). Moreover, their co-expression was associated to the presence of distant metastases at surgery and advanced TNM stage. Likewise, in a larger cohort of patients (n=200), a more comprehensive analysis of co-inhibitory receptors (PD-1, CTLA-4, TIM-3, LAG-3, and TIGIT) showed that a worse structural recurrence-free survival was associated with TIM-3, CTLA-4 expression, and PD-1/PD-L1 co-expression (213). Recently, Pozdeyev et al. showed that MTC is an immunologically active tumor: organized immune infiltration was observed in 49% of primary tumors and 90% of metastatic ones (214). CD8^+^ T as well as B cells are frequent observed while T regs were present in less than 5% of the non-tumoral cells.

Some anecdotal case of MTC treated with immunotherapy was reported. Del Rivero et al. described the case of a 61-year-old man affected by MTC who was enrolled on a clinical trial with heat-killed yeast-CEA vaccine, and thereafter on a phase I trial of a PD-L1 inhibitor (avelumab) (215). Interestingly, during both therapies the patient experienced a significant decrease of CTN, potential indication of structural response. On the other hand, Hedge et al. retrospectively reviewed patients harboring *RET-*mutated tumors who were enrolled in phase I clinical trials concerning immunotherapy or MKIs (216). They evaluated 32 patients with MTC of whom 28 were treated with MKIs and 4 with immunotherapy observing that the time to discontinuation for patients treated with MKIs was significantly longer than that of patients treated with immunotherapy alone (31.9 vs 8.2 months) (216). These results argued that immunotherapy alone could not be the first line choice in patients with metastatic MTC, but its combination with antiangiogenic (MKIs) or highly selective RET inhibitors could improve the disease control.

## Conclusions

Precision medicine is the new therapeutic frontier becoming always more precise. Over the years we learned to use drugs targeting several receptors that have profoundly changed the management of patients with advanced disease. In the last years, the increasing knowledge about clinical and molecular features of MTC has been leading to the development of drugs able to target specific mutated driver genes in a highly selective way. Future directions are towards drugs with high affinity for specific mutations of target genes and lower adverse events because of this very high selectivity.

However, despite this effort, about 30% of sporadic MTC have *RAS* mutations which are not druggable. Moreover, about 15% of MTC apparently have no mutations and therefore research should evaluate other directions to prevent these patients from remaining orphan to therapy. Radiometabolic and immunotherapy as well as other TKI with a broad spectrum of action need to be further studied and developed.

## Author Contributions

Conceptualization, AM and RE. Methodology, AM, CG, AP, and RE. Writing– original draft preparation, AM, CG, and AP. Writing– review and editing, all authors. Supervision, RE. All authors contributed to the article and approved the submitted version.

## Funding

This study has been supported by grants to RE from Associazione Italiana per la Ricerca sul Cancro (AIRC, Investigator grant 2018, project code 21790. Title: NEW INSIGHTS IN THE GENETIC PROFILE OF MEDULLARY THYROID CARCINOMA), Agenzia Italiana del Farmaco (AIFA, project code AIFA2016- 02365049. Title: Circulating microRNAs and DNA (cfDNA) as novel biomarkers for diagnostic, prognostic, and therapeutic use in Medullary Thyroid Carcinoma).

## Conflict of Interest

RE is consultant for Eisai, Loxo, Ipsen and Lilly, AM is consultant for Lilly, but the content of this paper was not influenced by this activity.

The remaining authors declare that the research was conducted in the absence of any commercial or financial relationships that could be construed as a potential conflict of interest.

## Publisher’s Note

All claims expressed in this article are solely those of the authors and do not necessarily represent those of their affiliated organizations, or those of the publisher, the editors and the reviewers. Any product that may be evaluated in this article, or claim that may be made by its manufacturer, is not guaranteed or endorsed by the publisher.
